# Polypharmacy in Elderly Patients Receiving First-Line Treatment for Pancreatic Adenocarcinoma: Analysis of Potential Drug–Drug Interactions and Outcome

**DOI:** 10.32604/or.2026.076944

**Published:** 2026-05-21

**Authors:** Stefano Vecchia, Rebecca Chinelli, Arianna Orcesi, Chiara Citterio, Alessandra Riva, Elena Orlandi

**Affiliations:** 1 Department of Pharmacy, Piacenza Hospital, Azienda USL of Piacenza, Piacenza, Italy; 2 Innovation, Research and Quality Unit, Piacenza Hospital, Azienda USL of Piacenza, Piacenza, Italy; 3 Department of Oncology-Hematology, Piacenza Hospital, Azienda USL of Piacenza, Piacenza, Italy

**Keywords:** Drug-drug interaction, pancreatic adenocarcinoma, pharmacology, oncology

## Abstract

**Objectives:** Pancreatic ductal adenocarcinoma (PDAC) is a leading cause of cancer-related mortality and mainly affects older adults, who frequently experience polypharmacy. While systemic therapy may improve outcomes in selected older patients, the use of multiple drugs increases the risk of potential drug–drug interactions (pDDIs). This study aimed to evaluate the prevalence and characteristics of pDDIs in older patients with PDAC receiving first-line systemic therapy and their potential impact on clinical outcomes. **Methods:** We conducted a retrospective single-center study including patients aged ≥ 75 years with PDAC who initiated first-line systemic therapy between December 2011 and January 2023. Prescription data were retrieved from institutional and outpatient pharmacy databases, and all concomitant medications were recorded. pDDIs were assessed using Intercheck Web and classified as A (minor), B (moderate), C (major but manageable), or D (contraindicated). Patients (n = 140) were stratified according to the number of active pharmaceutical ingredients (APIs) at treatment initiation: <5 (n = 53), 5–20 (n = 43), and >20 (n = 44). **Results:** A higher number of APIs was significantly associated with an increased burden of pDDIs, including A, B, C, and D interactions (all *p* < 0.001), with patients receiving >20 APIs showing the highest prevalence of clinically relevant interactions. Overall, 70% of patients had at least one pDDI, but no significant differences were observed in 1-year overall survival, access to second-line therapy, or chemotherapy type across API groups. **Conclusion:** Polypharmacy significantly increases the risk of pDDIs in older patients with PDAC, but this did not translate into differences in 1-year survival or access to subsequent therapy.

## 1 Introduction

Metastatic pancreatic ductal adenocarcinoma (mPDAC) is one of the most aggressive malignancies and is associated with a highly unfavorable prognosis [1]. Median survival in patients with mPDAC is generally less than one year, with a five-year mortality rate approaching 95–97% [2]. At disease onset, patients often present with poor performance status and significant weight loss, which is commonly linked to cancer-related cachexia [3]. Nausea and vomiting are frequent symptoms, typically resulting from local tumor infiltration that may compress the gastrointestinal tract [4]. In addition, a high metastatic burden further contributes to the deterioration of the patient’s overall clinical condition [5].

Common first-line treatment options include chemotherapy regimens such as NALIRIFOX, FOLFIRINOX, and nab-paclitaxel plus gemcitabine (Nab-PG) [6,7,8,9]. However, the use of these therapies in older adults presents specific challenges because of drug–drug interactions and a higher incidence of treatment-related toxicity. FOLFIRINOX, which combines oxaliplatin, irinotecan, fluorouracil, and leucovorin, has demonstrated a significant survival benefit compared with gemcitabine, but it is associated with substantial toxicity, including neutropenia, diarrhea, fever, peripheral neuropathy, and hepatotoxicity [6]. Nab-PG is associated with adverse events such as neutropenia, peripheral neuropathy, and thrombocytopenia [7], whereas gemcitabine monotherapy is generally better tolerated than combination therapy [6,7,8].

Although older adults account for a substantial proportion of patients in real-world practice—24.7% are diagnosed between ages 75 and 84, and 12.1% are over 80 years [10]—clinical trial data in this population remain limited. Advanced age is often an exclusion criterion in prospective studies [9], and the few available observational studies are fragmented and frequently focused on specific aspects such as dose modifications [11]. This underscores the need for additional prospective research to better define optimal treatment strategies for older adults with metastatic pancreatic cancer. The limited retrospective evidence comparing monotherapy with combination regimens in patients aged ≥ 75 years suggests that combination chemotherapy does not consistently provide a clear efficacy advantage over monotherapy and is associated with a higher incidence of adverse events [12,13].

Drug–drug interactions (DDIs) represent a major concern in oncology because polypharmacy is common and can compromise both the efficacy and the safety of cancer treatment. Patients routinely receive antineoplastic agents, supportive care medications, and drugs for comorbid conditions, leading to an increased risk of potential DDIs (pDDIs) [14,15,16]. Several studies have reported a high prevalence of pDDIs in oncology, most of which are of moderate severity and involve anticancer agents together with supportive therapies. The implementation of computerized prescribing systems with alert functionalities has helped reduce the number of clinically relevant interactions compared with earlier reports [17,18,19].

The risk of DDIs does not increase linearly but instead rises almost exponentially as the number of medications increases. In older adults without cancer, the probability of adverse drug reactions (ADRs) increases from 13% with two medications, to 58% with five, and up to 82% with seven or more [20]. In older adults with cancer, clinical frailty and comorbidities further magnify vulnerability. In a multicenter cohort of 718 patients (mean age 77 years), each additional medication was associated with a 39% increase in the risk of major pDDIs and a 12% increase in interactions involving anticancer treatments; notably, 61% of patients were already on polypharmacy (≥5 medications) at the initiation of chemotherapy [21].

Observational studies and systematic reviews have shown that, although combination chemotherapy may improve survival compared with monotherapy, it also carries a higher risk of severe toxicity, requiring careful assessment of the risk–benefit balance [22]. A retrospective study conducted at our center in late-elderly patients (≥75 years) with mPDAC confirmed these findings: combination therapy improved overall and progression-free survival at the cost of increased grade ≥ 3 adverse events [13]. In this context, tools such as comprehensive geriatric assessment (CGA) are recommended to guide therapeutic decision-making in older cancer patients, helping to balance treatment efficacy and tolerability [23].

In light of these considerations, the present study aims to investigate the prevalence and characteristics of DDIs in older cancer patients, with a specific focus on individuals with advanced PDAC enrolled in the ElderPanc study [13]. The goal is to identify key risk factors and determine whether the presence of pDDIs influenced clinical outcomes.

## 2 Materials and Methods

### 2.1 Study Design and Population

We conducted a retrospective, single-center observational study in patients aged ≥ 75 years with PDAC who initiated first-line systemic therapy between 01 December 2011 and 01 January 2023. Inclusion criteria included participation in the ElderPanc study [13] and the use, during the observation period, of at least one concomitant non-oncologic medication in combination with chemotherapy. Patients without concomitant non-oncologic medications and those with unavailable prescription data were excluded from the analysis. Patients were followed from the initiation of first-line systemic therapy until death or the last available follow-up. The primary outcome was the prevalence and distribution of pDDIs. Secondary outcomes included one-year overall survival and access to second-line therapy, described across groups stratified by the number of APIs.

The study was approved by the Institutional Review Board of Piacenza Hospital and by the Area Vasta Emilia Nord Ethics Committee (code 746/2023/OSS/AUSLPC) and was conducted in accordance with the Declaration of Helsinki. Written informed consent was obtained from each patient, when available (for deceased or non-contactable patients, data processing was performed in accordance with Italian data protection regulations), covering information on the study purpose, risks, benefits, and confidentiality of data. The reporting of this study adheres to the STROBE guidelines [24]. All datasets were complete for the main variables and no imputation was required. To limit selection bias, all consecutive eligible patients meeting the inclusion criteria during the study period were included. All patient information was pseudonymized to ensure confidentiality. A total of 140 patients met the inclusion criteria and were included in the analysis.

For each enrolled patient, prescription data for the treatment period were extracted from two institutional sources: the oncology prescribing and direct-distribution system for antineoplastic therapies and discharge medications (CCE-Log80^®^), and the outpatient pharmaceutical dispensing database of the territorial pharmacy service. Records from the two sources were linked using a unique patient identifier assigned within the institutional databases, enabling accurate matching of prescriptions across datasets. Data were organized by patients and by dispensing date. Active pharmaceutical ingredients (APIs) were considered at risk of interaction if the quantities dispensed were compatible with continuous use of the identified therapies—defined as at least one month of treatment per dispensing episode and consistent retrieval throughout the chemotherapy course.

### 2.2 Assessment of Drug–Drug Interactions

Potential drug–drug interactions were assessed and categorized using INTERCheck-web version 2.3.0 [25,26], a computerized clinical decision support system that classifies interactions according to their clinical relevance as follows: class D (contraindicated: combinations to be avoided); class C (major: combinations requiring close monitoring due to the potential for severe clinical consequences or treatment failure); class B (moderate: combinations requiring dosage adjustment and/or therapeutic drug monitoring); and class A (minor: combinations with no known clinical relevance). For qualitative analysis, APIs were grouped by pharmacological class within each pDDI risk category. All datasets were complete, with no missing values for the main variables.

### 2.3 Statistical Analysis

After identifying all medications taken by the patients and considering the ElderPanc study subpopulations (monotherapy vs. polytherapy), patients were categorized into three groups based on the number of concurrent medications: <5 medications, 5 to 20 medications, and more than 20 medications. Comparisons between categorical variables (including the distribution of potential drug–drug interactions, pDDIs, across medication groups) were conducted using the chi-square test or Fisher’s exact test, as appropriate. Continuous variables were analyzed using the Kruskal–Wallis test due to their non-normal distribution. When global comparisons were statistically significant, post hoc analyses with Bonferroni correction were performed to account for multiple testing.

Descriptive statistics were used to summarize patient demographics, clinical characteristics, and baseline prescribing patterns. Continuous variables are reported as medians with interquartile ranges (IQRs), while categorical variables are presented as absolute frequencies and percentages.

All statistical tests were two-sided, and a *p*-value < 0.05 was considered statistically significant. Analyses were performed using RStudio, version 3.6.0. Missing data were limited and are reported in the tables as “not reported”. No imputation methods were applied, and available-case analysis was performed for each variable.

## 3 Results

**Figure 1:**
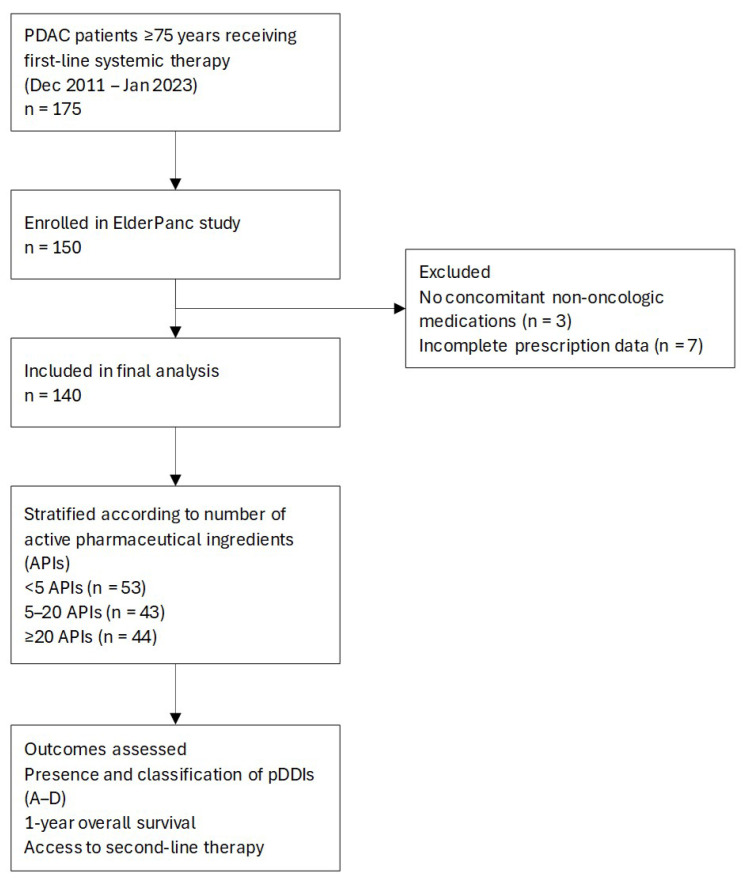
**Study flow diagram of patient selection and stratification.** PDAC = pancreatic ductal adenocarcinoma; APIs = active pharmaceutical ingredients; pDDIs = potential drug–drug interactions.

A total of 140 patients were included (Fig. 1) (64 [45.71%] receiving monotherapy and 76 [54.29%] receiving combination chemotherapy), as shown in Table 1.

The most represented age group was 75–79 years (53.6%), with no significant differences between groups. Females accounted for 49.3% of the study population. Most patients had a BMI ≥ 20 kg/m^2^ (85%) and an Eastern Cooperative Oncology Group performance status (ECOG-PS) of 0–1 (89.3%), with significantly different distributions between groups (81.2% in the monotherapy group vs. 96.1% in the combination-therapy group, *p* = 0.030).

The most common tumor location was the pancreatic head (52.1%). Overall, 8.4% of patients presented with metastatic disease at baseline, and nearly 30% had a metastatic burden > 1. The liver was the predominant metastatic site (52.9%). More than half of the cohort (53.6%) had a baseline CA19-9 level ≥ 200 U/mL. No significant differences in prior treatments (chemotherapy, radiotherapy, or surgery) were observed between the monotherapy and combination-therapy groups.

A dose reduction was applied in 93.6% of cases, with no significant difference in the overall distribution between groups. However, patients receiving a dose reduction ≥ 80% differed significantly between cohorts (75.3% in the combination-therapy group vs. 56.9% in the monotherapy group, *p* = 0.041). Comorbidities affected 82.1% of patients; diabetes was notably more prevalent in those receiving monotherapy (63.3% vs. 42.4%, *p* = 0.043).

Patients were additionally stratified into three groups based on the number of concomitant APIs: <5 APIs (n = 53, 37.86%), 5–20 APIs (n = 43, 30.71%), and >20 APIs (n = 44, 31.43%) (Table 2).

**Table 1:** Baseline characteristics of the patients enrolled in the study.

Characteristics	Patients (n = 140, 100%)	Monochemotherapy (n = 64, 45.71%)	Polichemotherapy (n = 76, 54.29%)	*p*-Value
Age (years)				
75–79, n (%)	75 (53.6)	30 (46.9)	45 (59.2)	0.261
80–84, n (%)	52 (37.1)	26 (40.6)	26 (34.2)	
>85, n (%)	13 (9.3)	8 (12.5)	5 (6.6)	
Sex				
Female, n (%)	69 (49.3)	30 (46.9)	39 (51.3)	0.723
BMI n (%)				
<20 kg/m^2^, n (%)	21 (15.0)	11 (17.2)	10 (13.2)	0.669
≥20 kg/m^2^, n (%)	119 (85.0)	53 (82.2)	66 (86.8)	
PS n (%)				
0–1, n (%)	125 (89.3)	52 (81.2)	73 (96.1)	0.030
>1, n (%)	10 (7.1)	8 (12.5)	2 (2.6)	
Not reported, n (%)	5 (3.6)	4 (6.3)	1 (1.3)	
Site				
Head, n (%)	73 (52.1)	32 (50.0)	41 (53.9)	0.772
Body, n (%)	51 (36.4)	19 (29.7)	31 (42.1)	0.473
Tail, n (%)	25 (17.9)	9 (14.1)	16 (21.1)	0.646
Stage				
III, non-resectable, (%)	26 (18.6)	11 (17.2)	15 (19.7)	0.068
IV, n (%)	114 (81.4)	53 (82.8)	61 (80.3)	
Metastatic burden				
1, n (%)	75 (53.6)	32 (50.0)	43 (56.6)	0.349
>1, n (%)	39 (27.9)	21 (32.5)	18 (23.7)	
Metastasis site				
Lung, n (%)	30 (21.4)	15 (23.4)	15 (19.7)	0.436
Liver, n (%)	74 (52.9)	34 (53.1)	40 (52.6)	0.408
Peritoneal, n (%)	33 (23.6)	14 (21.9)	19 (25.0)	>0.999
Other, n (%)	22 (15.7)	13 (20)	9 (11.8)	0.127
ca19.9				
<200, n (%)	40 (28.6)	14 (21.9)	26 (34.5)	0.462
≥200 U/mL, n (%)	75 (53.6)	33 (51.6)	42 (55.3)	
Not reported, n (%)	25 (17.8)	17 (26.5)	8 (10.5)	
Previous chemotherapy, n (%)	22 (15.7)	8 (12.5)	14 (18.4)	0.630
Previous radiotherapy, n (%)	7 (5.0)	3 (4.7)	4 (5.3)	>0.999
Previous surgery, n (%)	21 (15.0)	7 (10.9)	14 (18.4)	0.446
Dose reduction, n (%)	131 (93.6)	58 (90.6)	73 (96.1)	0.338
Dose reduction ≥ 80%, n (%)	88 (67.2)	33 (56.9)	55 (75.3)	0.041
Comorbidity	115 (82.1)	49 (76.6)	66 (86.8)	0.836
Cardiac, n (%)	36 (31.3)	16 (32.7)	20 (30.3)	0.948
Pulmonary, n (%)	12 (10.4)	7 (14.3)	5 (7.6)	0.392
Liver, n (%)	7 (6.1)	4 (8.2)	3 (4.5)	0.683
Diabetes, n (%)	59 (51.3)	31 (63.3)	28 (42.4)	0.043
Hypertension, n (%)	71 (61.7)	32 (65.3)	39 (59.1)	0.701
≥3 comorbidities n (%)	18 (15.7)	8 (16.3)	10 (15.2)	>0.999

Note: BMI: Body Mass Index; PS: Performance Status.

**Table 2:** Patient stratification according to the number of concomitant active pharmaceutical ingredients.

Variable	Total n = 140	APIs < 5 n = 53 (37.86%)	APIs 5–20 n = 43 (30.71%)	APIs > 20 n = 44 (31.43%)	*p*-Value
Monotherapy n (%)	61 (43.6)	24 (45.3)	16 (37.2)	21 (47.7)	0.583
Combination therapy n (%)	79 (56.4)	29 (54.7)	27 (62.8)	23 (52.3)	
Any pDDI present n (%)	98 (70)	14 (36.4)	40 (93)	44 (100)	<0.001
Type A interactions n (%)	63 (45)	3 (5.7)	17 (39.5)	43 (97.7)	<0.001
Type A median [IQR] (range)	0 [0–3] (0–48)	0 [0–0] (0–1)	0 [0–1] (0–4)	8.5 [3–15] (0–48)	<0.001
Type B interactions n (%)	81 (57.9)	3 (5.7)	35 (81.4)	43 (97.7)	<0.001
Type B median [IQR] (range)	1 [0–31.5] (0–691)	0 [0–0] (0–2)	3 [1–8] (0–391)	71.5 [38.5–126.75] (0–391)	<0.001
Type C interactions n (%)	84 (60)	8 (15.1)	33 (76.7)	43 (97.7)	<0.001
Type C median [IQR] (range)	1 [0–23] (0–395)	0 [0–0] (0–2)	2 [1–5] (0–19)	42.5 [28.25–75] (0–359)	<0.001
Type D interactions n (%)	51 (36.4)	2 (3.8)	12 (27.9)	37 (84.1)	<0.001
Type D median [IQR] (range)	0 [0–1] (0–46)	0 [0–0] (0–1)	0 [0–1] (0–6)	2.5 [1–6] (0–46)	<0.001
1-year OS median [IQR] (range)	200 [115–345.2] (14–365)	186 [142–285] (14–365)	215 [116–365] (18–365)	218.5 [109.8–365] (31–365)	0.567
Second-line therapy n (%)	49 (35)	15 (28.3)	17 (29.5)	17 (38.6)	0.430

Note: APIs: Active Pharmaceutical Ingredients; pDDI: Potential Drug–Drug Interaction; IQR: Interquartile Range.

The distribution of chemotherapy regimens was similar across the three subgroups, with no significant differences between monotherapy and combination chemotherapy.

Drug–drug interactions were overall frequent (98/140 patients, 70%), with a clear increasing gradient according to the number of APIs: from 36.4% in patients taking <5 medications, to 93% in those taking 5–20 APIs, and up to 100% in those taking >20 APIs (*p* < 0.001).

Analysis by interaction category confirmed the same pattern. Type A interactions were present in 45% of patients, with the median increasing from 0 (0–0) in the <5 API group to 8.5 (3–15) in the >20 API group (*p* < 0.001). Similarly, type B interactions were observed in 57.9% of patients, with a median of 1 (0–31.5), rising significantly to 71.5 (38.5–126.75) in the >20 API group (*p* < 0.001). Type C interactions were present in 60% of cases, with a median of 42.5 (28.25–75) in the >20 API group compared with 0 (0–0) in the <5 API group (*p* < 0.001). Type D interactions also showed an increasing frequency (36.4% overall, up to 84.1% in the >20 API group), with significant differences across groups (*p* < 0.001).

The median one-year overall survival was 200 days (115–345.2), with no statistically significant differences among the three subgroups. Similarly, 35% of patients received second-line treatment, with no significant differences across groups.

A total of 4947 pDDIs were identified among the enrolled patients, including 282 type A interactions (5.7%), 2539 type B interactions (51.3%), 1884 type C interactions (38.1%), and 242 type D interactions (4.9%). Given the large number of potential interactions identified, only the most frequent drug combinations within each interaction category and their potential pharmacological effects are summarized in Table 3. All interactions were identified as pDDIs based on computerized screening, and no systematic assessment of clinically manifest DDI-related adverse events was performed.

**Table 3:** Main interactions identified.

Grade	Interacting Classes	Description	N (%)
A	Beta-blockers–Corticosteroids	Corticosteroids may reduce the antihypertensive effect of beta-blockers due to sodium and water retention [27]	52 (18.4)
A	Calcium-channel blockers (dihydropyridines)–Corticosteroids	Corticosteroids may attenuate the antihypertensive effect of calcium-channel blockers [27]	33 (11.7)
A	Corticosteroids–Sartans (ARBs)	Possible antagonism of the antihypertensive effect of ARBs by corticosteroids [27]	30 (10.6)
A	ACE inhibitors–Corticosteroids	Corticosteroids may reduce the antihypertensive effect of ACE inhibitors [27]	30 (10.6)
A	Corticosteroids–Diuretics	Additive risk of hypokalemia (mineralocorticoid effect + diuretic kaliuresis) [28]	24 (8.5)
A	Fibrates–Statins	Increased risk of myopathy/rhabdomyolysis due to additive toxicity [29]	17 (6.0)
B	Beta-blockers–Corticosteroids	Reduced antihypertensive response; monitor blood pressure and edema [27]	133 (5.2)
B	Beta-blockers–Calcium-channel blockers (dihydropyridines)	Additive risk of hypotension; less problematic vs. non-DHP CCBs [30]	125 (4.9)
B	Beta-blockers–NSAIDs	NSAIDs reduce the antihypertensive effect of beta-blockers via renal prostaglandin inhibition [31]	112 (4.4)
B	Diuretics–PPIs	Increased risk of hypomagnesemia/hypokalemia with arrhythmic consequences [32]	96 (3.8)
B	Antiplatelet agents–PPIs	Clopidogrel + omeprazole/esomeprazole: reduced activation due to CYP2C19 inhibition [33]	96 (3.8)
B	Antiplatelet agents–Beta-blockers	High-dose ASA may reduce antihypertensive efficacy; irrelevant at low antiplatelet doses [34]	94 (3.7)
C	Corticosteroids–NSAIDs	Increased risk of gastrointestinal ulcer and bleeding [35]	86 (4.6)
C	ACE inhibitors–NSAIDs	Risk of renal failure (“triple whammy”) and loss of blood pressure control [36]	79 (4.2)
C	ACE inhibitors–Sartans (ARBs)	Risk of hyperkalemia and renal failure without additional benefit [37]	71 (3.8)
C	Antiplatelet agents–NSAIDs	Additive bleeding risk, especially gastrointestinal [35]	62 (3.3)
C	PPIs–Macrolides	Possible pharmacokinetic interaction and additive QT prolongation [38]	61 (3.2)
C	Antiplatelet agents–Corticosteroids	Increased risk of gastrointestinal bleeding [35]	61 (3.2)
D	Antiarrhythmics–Macrolides	Additive risk of QT prolongation and torsades de pointes [39]	12 (5.0)
D	Fluoroquinolones–Macrolides	Both prolong QT → increased arrhythmic risk [40]	10 (4.1)
D	Beta-blockers–Beta2-agonist bronchodilators	Pharmacodynamic antagonism → risk of bronchospasm [41]	9 (3.7)
D	Antiarrhythmics–Fluoroquinolones	Risk of ventricular arrhythmias due to QT prolongation [40]	8 (3.3)
D	SSRIs–Fluoroquinolones	Additive QT risk; possible serotonin syndrome in some cases [42]	7 (2.9)
D	SSRIs–Prokinetics/Antiemetics	Metoclopramide + SSRIs: serotonin syndrome; Ondansetron + SSRIs: QT prolongation [42]	7 (2.9)
D	Macrolides–Opioids	Macrolides inhibit CYP3A4 → increased oxycodone/methadone levels and QT risk [43]	6 (2.5)
D	Antiepileptics–Macrolides	Macrolides inhibit CYP3A4 → ↑ carbamazepine/phenytoin levels with neurotoxicity [44]	6 (2.5)

Note: ARBs: Angiotensin II Receptor Blockers; non-DHP CCBs: Non–Dihydropyridine Calcium Channel Blockers; NSAIDs: Nonsteroidal Anti-Inflammatory Drugs; PPIs: Proton Pump Inhibitors; SSRIs: Selective Serotonin Reuptake Inhibitors.

Regarding type A interactions, the most frequent combinations involved corticosteroids combined with cardiovascular drugs, including beta-blockers, calcium-channel blockers, sartans, ACE inhibitors, and diuretics, as well as fibrates with statins.

Type B interactions included 291 different combinations, with the most common again involving cardiovascular drugs in combination with corticosteroids. Other common patterns included beta-blockers with calcium-channel blockers or NSAIDs, diuretics with proton pump inhibitors (PPIs), and antiplatelet agents with PPIs or beta-blockers.

Type C interactions were distributed across 303 combinations and involved a wide range of drug classes. The most frequent combinations included corticosteroids with NSAIDs, ACE inhibitors with NSAIDs or sartans, antiplatelet agents with NSAIDs or corticosteroids, and PPIs with macrolides.

Finally, type D interactions, distributed across 101 combinations, represented the most critical associations. These mainly involved QT-prolonging drug combinations, beta-blockers with β2-agonists, and macrolide-mediated interactions with drugs such as opioids or antiepileptics.

## 4 Discussion

In this study, we analyzed the prevalence and characteristics of potential drug–drug interactions in a cohort of 140 patients aged ≥ 75 years with advanced pancreatic ductal adenocarcinoma enrolled in the ElderPanc study. Our findings show a high frequency of pDDIs (70% overall), with a prevalence directly proportional to the number of active pharmaceutical ingredients taken. In patients with substantial polypharmacy (>20 concomitant medications), interactions were ubiquitous, and more than 80% of patients had at least one type D interactions, considered contraindicated.

These results are consistent with existing literature, which reports a high prevalence of pDDIs in cancer patients, although rates vary depending on clinical setting and methodological approach. Earlier studies reported prevalence estimates between 27% and 58% [14,15], whereas more recent cohorts have shown rates between 70% and 88% [16,20]. The prevalence observed in our population is therefore higher than that described in earlier historical cohorts but aligns with contemporary findings, likely reflecting the older age of patients, the greater burden of concomitant medications, and the use of more sensitive computerized screening tools.

A central finding of our analysis is the strong association between the total number of medications and the prevalence of pDDIs. The frequency of pDDIs increased almost exponentially, becoming universal in patients receiving > 20 medications. This trend is consistent with previous observations: Riechelmann et al. and van Leeuwen et al. reported a marked increase in interaction risk with each additional drug [14,15], while Ramsdale et al., in a large geriatric oncology cohort, quantified a 39% increase in major pDDIs and a 12% increase in interactions involving anticancer drugs for each additional medication [21]. Moreover, the total number of interactions identified in our study was high (4947). We examined medications taken at diagnosis and during the initiation of chemotherapy and observed a substantial pharmacological burden, often related to treatments commonly used to manage symptoms of pancreatic cancer (such as hypertension, hyperglycemia, and pain). This highlights the need for closer monitoring of pre-diagnosis prescribing escalation to protect patients from potential pDDIs.

The distribution of pharmacological classes most frequently involved also mirrors previous reports. In our cohort, the most common combinations included corticosteroids with cardiovascular medications (β-blockers, ACE inhibitors, sartans, calcium-channel blockers, and diuretics), NSAIDs, proton pump inhibitors, and macrolide/fluoroquinolone antibiotics among the most severe interactions. These pharmacological clusters have been consistently identified as particularly relevant in oncology [14,15,16,21,45], underscoring the persistence of well-known critical areas that remain challenging to mitigate in routine clinical practice.

From a mechanistic perspective, most pDDIs identified in our cohort were pharmacodynamic. These interactions mainly reflected additive or antagonistic effects on physiological systems, including blood pressure regulation, electrolyte balance, bleeding risk, and cardiac repolarization. As such, they are not mediated by specific metabolic pathways and are generally manageable through appropriate clinical monitoring and dose adjustment. Pharmacokinetic interactions were less frequent but may be more clinically relevant despite their lower frequency, as they can significantly alter drug exposure. In our cohort, these were primarily related to cytochrome P450–mediated mechanisms, particularly involving CYP3A4 and CYP2C19. Representative examples include reduced clopidogrel activation with proton pump inhibitors through CYP2C19 inhibition and increased exposure to drugs such as opioids or carbamazepine when co-administered with macrolides due to CYP3A4 inhibition. Overall, these findings suggest that while pharmacodynamic interactions predominate in this population, pharmacokinetic interactions require particular attention because of their potential impact on drug concentrations and toxicity. This may partly explain the lack of association observed between pDDI burden and survival outcomes in our study, as most interactions were potentially manageable in clinical practice.

Importantly, despite the high incidence of pDDIs, no significant differences emerged in one-year overall survival or access to second-line treatment across groups stratified by the number of concomitant medications. This finding is noteworthy because, in the available literature, the relationship between pDDIs—considered in a global sense (number or severity)—and oncologic outcomes has rarely been investigated directly, and published results are limited and inconclusive [14,16,21]. Most studies have described the prevalence of pDDIs or assessed their potential clinical implications in terms of toxicity or treatment management, without demonstrating a consistent association with OS or PFS. It is plausible that, in advanced pancreatic cancer, the intrinsic aggressiveness of the disease remains the dominant determinant of prognosis, thereby attenuating any observable effect of pDDIs on survival outcomes. Furthermore, assessing pDDIs without systematic documentation of related clinical adverse events limits the ability to capture their most immediate consequences, such as iatrogenic toxicities or reduced treatment adherence.

In daily oncology practice, recognizing these pharmacodynamic interactions may guide treatment decisions by encouraging closer clinical monitoring, adjustment of concomitant medications, or substitution of high-risk drug combinations when feasible.

From a practical perspective, our findings reinforce the importance of implementing structured medication reconciliation processes and proactive pDDI assessment in older cancer patients. Integrating comprehensive geriatric assessment and involving clinical pharmacists are essential strategies to balance treatment efficacy and safety [23,46]. Interaction screening should not be viewed as a purely pharmacological exercise but as part of a broader multidisciplinary decision-making process. Identifying high pDDI burden at baseline may prompt closer collaboration between oncologists, geriatricians, and clinical pharmacists, particularly when selecting between monochemotherapy and combination regimens or when intensifying supportive care. In selected cases, awareness of high-risk interaction profiles may support proactive dose adjustments, deprescribing of non-essential medications, or alternative supportive strategies. The use of computerized prescribing support systems may help identify and prevent the most critical interactions, but their output requires clinically contextualized, multidisciplinary interpretation.

Our study has several limitations: its retrospective, single-center design; the relatively small sample size; and the lack of systematic data on clinical adverse events, which prevents establishing a causal relationship between pDDIs and clinical outcomes.

Prospective multicenter studies with larger cohorts and active monitoring of DDI-related adverse events are needed to clarify the clinical impact of pDDIs on oncologic outcomes and patient quality of life, and to better link pharmacological interactions with real-world clinical toxicity. In particular, a specific focus on pharmacist-led medication reconciliation at chemotherapy initiation may help assess whether targeted deprescribing or modification of high-risk drug combinations can reduce clinically relevant toxicities without compromising oncologic efficacy. Systematic implementation of screening tools, together with dedicated staff training and structured involvement of clinical pharmacists, may further improve medication safety in this particularly vulnerable population.

## Data Availability

The data that support the findings of this study are available from the Corresponding Author, [EO], upon reasonable request.

## Abbreviations

pDDIs	Potential drug-drug interactions
PDAC	Pancreatic ductal adenocarcinoma
mPDAC	Metastatic pancreatic ductal adenocarcinoma
Nab-PG	Nab-paclitaxel plus gemcitabine
DDIs	Drug-drug interactions
ADRs	Adverse drug reactions
CGA	Comprehensive geriatric assessment
APIs	Active pharmaceutical ingredient
IQRs	Interquartile ranges
BMI	Body mass index
PS	Performance status
ECOG	Eastern cooperative oncology group
NSAIDs	Nonsteroidal anti-inflammatory drugs
ARBs	Angiotensin receptor blockers
PPIs	Proton-pump inhibitors
SSRIs	Selective serotonin reuptake inhibitors
